# Differences in Facial Expression Recognition Between Unipolar and Bipolar Depression

**DOI:** 10.3389/fpsyg.2021.619368

**Published:** 2021-07-14

**Authors:** Ma Ruihua, Zhao Meng, Chen Nan, Liu Panqi, Guo Hua, Liu Sijia, Shi Jing, Zhao Ke, Tan Yunlong, Tan Shuping, Yang Fude, Tian Li, Wang Zhiren

**Affiliations:** ^1^Peking University HuiLongGuan Clinical Medical School, Beijing Huilongguan Hospital, Beijing, China; ^2^Department of Neurosurgery, Sanbo Brain Hospital, Capital Medical University, Beijing, China; ^3^Zhumadian Psychiatric Hospital, Zhumadian, China; ^4^State Key Laboratory of Brain and Cognitive Science, University of the Chinese Academy of Sciences, Beijing, China; ^5^Department of Physiology, Faculty of Medicine, Institute of Biomedicine and Translational Medicine, University of Tartu, Tartu, Estonia

**Keywords:** unipolar depression, bipolar depression, rapid facial expression recognition, react time, cognitive function

## Abstract

**Purpose:**

To explore the differences in facial emotion recognition among patients with unipolar depression (UD), bipolar depression (BD), and normal controls.

**Methods:**

Thirty patients with UD and 30 patients with BD, respectively, were recruited in Zhumadian Second People’s Hospital from July 2018 to August 2019. Fifteen groups of facial expressions including happiness, sadness, anger, surprise, fear, and disgust were identified.

**Results:**

A single-factor ANOVA was used to analyze the facial expression recognition results of the three groups, and the differences were found in the happy-sad (*P* = 0.009), happy-angry (*P* = 0.001), happy-surprised (*P* = 0.034), and disgust-surprised (*P* = 0.038) facial expression groups. The independent sample *T*-test analysis showed that compared with the normal control group, there were differences in the happy-sad (*P* = 0.009) and happy-angry (*P* = 0.009) groups in patients with BD, and the accuracy of facial expression recognition was lower than the normal control group. Compared with patients with UD, there were differences between the happy-sad (*P* = 0.005) and happy-angry (*P* = 0.002) groups, and the identification accuracy of patients with UD was higher than that of patients with BD. The time of facial expression recognition in the normal control group was shorter than that in the patient group. Using happiness-sadness to distinguish unipolar and BDs, the area under the ROC curve (AUC) is 0.933, the specificity is 0.889, and the sensitivity is 0.667. Using happiness-anger to distinguish unipolar and BD, the AUC was 0.733, the specificity was 0.778, and the sensitivity was 0.600.

**Conclusion:**

Patients with UD had lower performance in recognizing negative expressions and had longer recognition times. Those with BD had lower accuracy in recognizing positive expressions and longer recognition times. Rapid facial expression recognition performance may be as a potential endophenotype for early identification of unipolar and BD.

## Introduction

Mood disorder is also called affective mental disorder, which refers to a group of diseases that are caused by various reasons and are characterized by significant and lasting changes in emotion or mood. Mood disorders include depression and bipolar disorder. In 2017, the World Health Organization (WHO) reported that depression, which affects 322 million people globally, is the second leading cause of the World’s medical burden. It is expected that by 2030, depression will become the most common disabling condition (Duric et al., 2018). Depression is a common disease with low mood, lack of interest, fatigue, and other core symptoms. It is a complex mental disorder caused by multiple factors, which seriously affects people’s health. It is generally believed to be related to social, environmental, and personal factors, but the specific pathogenesis is still unclear (Kang and Cai, 2017). Bipolar disorder, formerly known as manic depression, is characterized by alternating episodes or interweaving of mania, hypomania, and depression, and is called bipolar depression (BD) when in the depressed phase (Grande et al., 2016). However, BD is misdiagnosed as unipolar depression (UD) in up to 40% of patients due to its similarities, and those patients are negatively affected because they are not treated with mood stabilizers (Kessing et al., 2017). It is particularly important for determining biomarkers that distinguish UD and BD. Endophenotype is a highly heritable disease-associated risk symptom specific to the disease, independent of the clinical state of the disease. Discovery of unique endophenotypes for BD and UD may thus provide a new approach for the etiologies of each disorder and aid earlier detection and appropriate treatment. Cognitive abnormalities might be the most promising endophenotype of affective disorders (Miskowiak et al., 2016).

Facial emotion recognition is a special field of cognition which involves interpreting the emotions of others based on their facial expressions. Further, correctly recognizing facial expression is very important for normal communication and social functioning, but emotional signal recognition is disturbed in many mental diseases (Altamura et al., 2016; Jack et al., 2018). In the 19th century, Darwin stated that happiness, anger, sadness, and joy are the basic facial expressions of human beings through a long-term study on the facial expressions of humans and animals (Darwin et al., 1998). Since then, Ekman and Friesen proposed that human facial expressions include six basic emotions: happiness, sadness, anger, fear, aversion, and surprise. The six basic emotions have cross-cultural and inter-racial stability (Ekman et al., 1987). In 1978, with the development of the Facial Action Coding System, facial expression recognition became a hot topic in the field of psychology and psychiatry. Recent studies have shown that the deficit in facial expression recognition is related to depression disorders (Dalili et al., 2015). Moreover, it was found that there are emotional and cognitive dysfunctions in both UD and BD (Neves et al., 2015; Wu et al., 2017). Several studies have noted that the accuracy of emotional recognition in depressed patients generally tends to decrease. Previously, Beck (Copeland, 1970) proposed that negative self-evaluations, beliefs, and memories play a key role in depression. Since then, some studies have found that people with depression have a negative bias in facial expression recognition, and tend to interpret neutral faces as sad (Gur et al., 1992; Leppänen et al., 2004; Lee et al., 2016). In addition, other studies have found that people with depression identify happy faces less accurately, and were less likely than normal people to interpret neutral expressions as happy expressions or more sensitive to angry expressions (Murray, 2000; Zwick and Wolkenstein, 2017). At the same time, some studies have found that people with bipolar disorder have significantly reduced ability to recognize emotions in faces, especially in sad and fearful faces (Vederman et al., 2012; Samamé et al., 2015). Conversely, other studies have found that patients with bipolar disorder have a reduced ability to recognize happy expressions (Lawlor-Savage et al., 2014). Vederman et al. (2012) found that BD patients have a lower ability to recognize sad and fearful facial expressions than UD patients, which may be one of the characteristics of identifying bipolar disorder, though there are few studies on the difference between UD and BD patients in facial expression recognition. Based on research reports that cognitive dysfunction between UD and BD is different, the facial expression recognition ability of patients with UD and BD may also be different (Miskowiak et al., 2012). The purpose of this study is to provide a theoretical basis for clinical differentiation UD and BD by comparing their facial expression recognition differences.

## Materials and Methods

### Participants

#### Patient Group

There were 30 patients diagnosed with UD and 30 patients diagnosed with BD, all of which were outpatients or inpatients at Zhumadian Second People’s Hospital from July 2018 to August 2019. Enrolment criteria: (1) met the U.S. Diagnostic and Statistical Manual (Fourth Edition, DSM-IV, Fourth Edition) criteria for either a depression diagnosis, whether UD or BD; (2) HAMD17 ≥ 17 points, the number of depression episodes in patients with UD was more than two times, and follow-up was not performed after 8 weeks; (3) right-handed; (4) Han nationality; (5) aged 18–50 years; and (6) after a detailed explanation, the participant signed the informed consent form. Exclusion criteria: (1) combined with other mental disorders; (2) history of cerebral organic diseases, or history of craniocerebral injury, electrical shock treatment, or other serious physical diseases; (3) history of alcohol and substance abuse; (4) intellectual disability; (5) pregnant and lactating women; and (6) he or she has been taking antipsychotic drugs regularly for the past 2 months.

#### Control Group

Thirty healthy subjects in the community surrounding Zhumadian Second People’s Hospital during the same period were enrolled. Enrolment criteria: (1) never suffered from any mental disorder in the past; (2) matched the patient groups’ race, hands, age, gender, and education years; (3) HAMD17 < 7 points; (4) family history of mental disorders was negative; and (5) after understanding the entire experimental process, volunteered to participate in the test and signed an informed consent form, understood, and cooperated with the inspection. Exclusion criteria: (1) first-degree relatives have been diagnosed with mental illness; (2) history of cerebral organic diseases, or history of craniocerebral injury, electrical shock treatment, or other serious physical illnesses; (3) alcohol and substance abuse; (4) intellectual disability; and (5) pregnant and lactating women.

This study was reviewed and approved by the Ethics Committee of Beijing Huilongguan Hospital and the Ethics Committee of Zhumadian Psychiatric Hospital. All participants were informed of the content of the trials and the risks or benefits that may arise from participating in the study. Further, all participants signed informed consent forms.

### Emotion Recognition Task

The emotion recognition task was compiled and run with E-prime2.0 (Experimental Program Software). The experimental instrument was a Dell laptop computer. The display is a 16-inch (31 cm × 17.5 cm) built-in monitor with a refresh rate of 60 Hz and a resolution of 1,280 pixels × 800 pixels. The eyes of the subjects were placed about 60 cm from the center of the screen.

**FIGURE 1:**
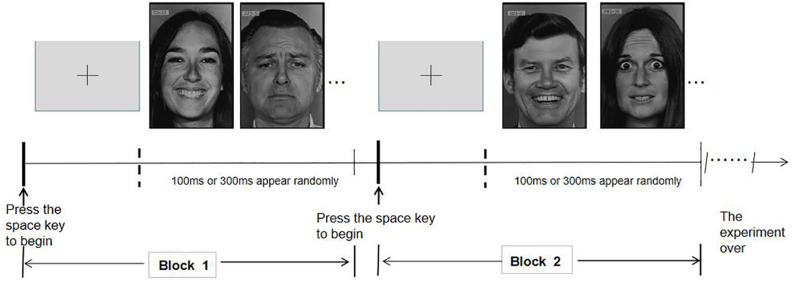
Graph of emotion recognition task.

Participants performed facial expression recognition tests. Ten models (six women and four men) were selected from the Ekman database. Each model had six unique facial expressions (happiness, sadness, fear, disgust, surprise, and anger), with a total of 60 pictures. In each block, there were 20 pictures displaying of two facial expressions. After the subjects pressed the “space” key, participants were presented with a fixation point “+” for 200 ms in the center of the screen. After the fixation point disappeared, they were randomly presented with a picture of a model’s emotional expression. The presentation time is 100 ms or 300 ms, which appears randomly. The task was to choose 1 or 2 of the two expression options to judge the expression presented. The participants were required to make judgments within the limited time of the image, or they would automatically skip to the next image. An expression without judgment was marked as wrong. The subjects were asked to identify 15 sets of facial expressions made up of six different facial expressions. As shown in Figure 1, in each set of the tests, for example, to identify happy and sad faces, 10 models had two random faces at 20 times in all. 1 corresponds to happiness, 2 to sadness, and the subjects judge and choose. After completion of one trial of the task, participants press the “space” key to start the next trial.

### Statistics

SPSS19.0 was used for statistical analysis, and the experimental data of each group were expressed by x ± s. Chi-square test was used for sex between groups; ANOVA and *post-hoc* LSD pairwise test were used to compare age and education level. Independent sample *t* test was used to compare the HAMD score, HAMA score, and YMRS score of the two patient groups. Bilateral test, *P* < 0.05 was considered statistically significant.

We use *d’* to represent the accuracy of facial expression recognition, that is, the measurement value of discriminative ability, which follows the signal detection theory, and uses the hit rate and false positive rate to estimate the recognition ability (Macmillan and Creelman, 2005). The single-factor ANOVA was used to analyze the *d’* values of the three groups of facial expressions. In order to evaluate the difference in facial expression recognition speed between the three groups, single-factor ANOVA was used to analyze the response time (RT) of the three groups of facial expression recognition.

## Results

### Clinical Data Analysis

Bipolar depression, UD, and normal control groups did not differ from each other in age (*F* = 1.88, *p* = 0.16), gender (*X*^2^ = 2.69, *p* = 0.26), and education (*F* = 3.00, *p* = 0.06). There was no significant difference in HAMD (*t* = 2.08, *p* = 0.95), HAMA (6.99, *p* = 0.40), and YMRS scores (*t* = 2.25, 0.32) between UD and BD (Table 1).

**TABLE 1:** Comparison of general demographic data and clinical symptom scores ($\bar{\text{x}}$ ± s).

	Unipolar (*n* = 30)	Bipolar (*n* = 30)	Control (*n* = 30)	t/F/ *X2*	*p*
Age (year, $\bar{\text{x}}$ ± s)*	28.30 ± 9.73	24.25 ± 9.03	28.45 ± 7.49	1.88	0.16
Gender (Case, man/women)^&^	11/19	13/17	12/8	2.69	0.26
Years of education (year, $\bar{\text{x}}$ ± s)*	10.57 ± 2.43	10.59 ± 2.91	12.32 ± 3.20	3.00	0.06
HAMD-17 (score, $\bar{\text{x}}$ ± s)	21.50 ± 6.63	21.59 ± 3.24	—	2.08	0.95
HAMA (score)	18.87 ± 5.54	20.67 ± 9.67	—	6.99	0.40
YMRS (score)	1.87 ± 1.70	2.96 ± 2.07	—	2.25	0.32

*represents analysis of variance, ^&^represents chi-square test, and the rest is *t* test.

*HAMD-17, Hamilton Depression Scale; HAMA, Hamilton Anxiety Scale; YMRS, Young’s Mania Scale.*

### Facial Expression Recognition Data Analysis

**FIGURE 2:**
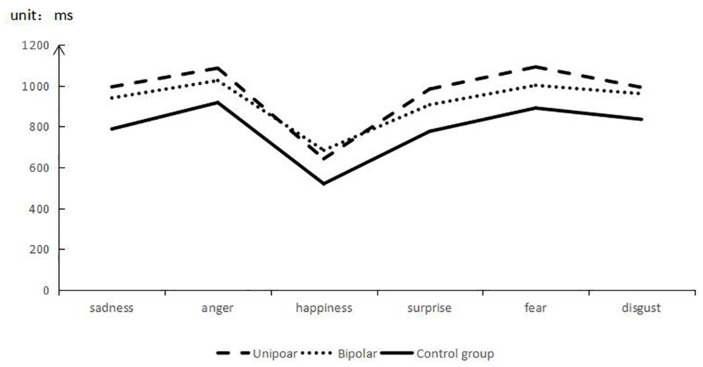
Accuracy of facial expression recognition.

Figure 2 shows that there are statistically significant differences in the recognition of happiness-sadness (*p* = 0.009) and happiness-anger (*p* = 0.009) between the control group and group of patients with BD, and the recognition accuracy of the control group was higher. Comparing UD group to BD group, there is difference in happiness-sadness (*p* = 0.005) and anger-happiness (*p* = 0.002), and the recognition accuracy of UD group was better than that of BD group (see Table 2 for details).

**TABLE 2:** Facial expressions recognize *d’* values ($\bar{\text{x}}$ ± s).

	Unipolar	Bipolar	Control group	*P*
Sa-An	1.36 ± 0.95	1.79 ± 1.57	1.97 ± 0.86	0.163
Sa-Ha	6.75 ± 2.67^a^	4.52 ± 3.95^b^	6.53 ± 2.82	0.009
Sa-Su	4.36 ± 3.11	3.78 ± 3.02	5.76 ± 2.93	0.074
Sa-Fe	2.94 ± 2.42	2.16 ± 1.63	2.99 ± 1.94	0.261
Sa-Di	2.25 ± 1.61	2.09 ± 2.05	2.83 ± 1.49	0.321
An-Ha	6.37 ± 2.79^a^	4.03 ± 2.55^b^	6.60 ± 2.71	0.001
An-Su	6.37 ± 2.21	4.03 ± 2.27	6.60 ± 2.61	0.435
An-Fe	1.31 ± 1.10	1.80 ± 2.13	2.32 ± 1.61	0.097
An-Di	0.25 ± 0.61	0.26 ± 0.69	0.48 ± 0.86	0.469
Ha-Su	5.28 ± 2.77	3.98 ± 2.88	6.10 ± 2.82	0.034
Ha-Fe	6.01 ± 2.83	4.52 ± 3.01	6.37 ± 2.75	0.056
Ha-Di	6.23 ± 2.76	5.18 ± 3.18	6.12 ± 2.79	0.347
Su-Fe	1.05 ± 0.87	1.00 ± 0.97	1.31 ± 0.70	0.454
Su-Di	4.36 ± 3.17	3.80 ± 3.04	6.02 ± 2.90	0.038
Fe-Di	2.73 ± 2.50	2.76 ± 2.68	4.00 ± 2.99	0.183

**P* represents the value of statistical analysis among the three groups.*

*^*a*^represents a statistical difference between patients with UD and BD.*

*^*b*^represents a statistical difference between patients with BD and the control group.*

**FIGURE 3:**
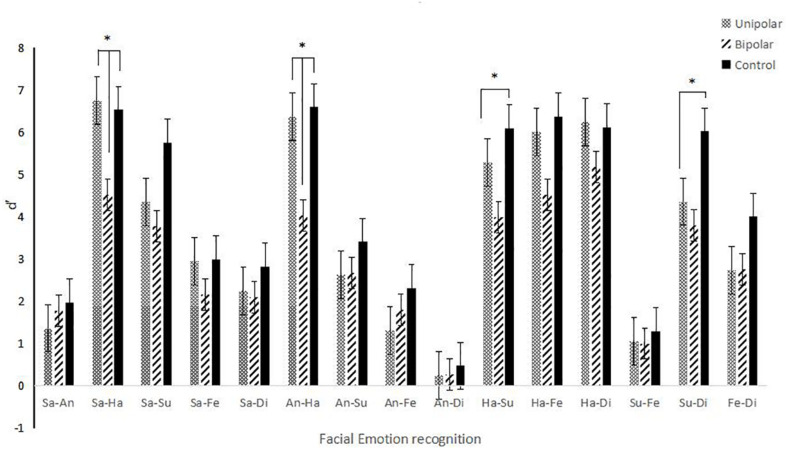
React time of facial expression recognition. Note: “*” represents *p* < 0.05. “__” represents differences between two groups or among three groups.

The RTs of the facial expression among the three groups were different, and the RT in the normal control group was shorter than that of the patient groups (Figure 3). Compared to the two patient groups, BD group took a longer time to recognize happy expressions, while UD group took a longer time to recognize negative and neutral expressions (see Table 3 for details).

**TABLE 3:** Response time among the three groups (ms).

	Unipolar	Bipolar	Control	*p*
Sad	993.61 ± 203.61	939.28 ± 197.80	787.46 ± 176.83	0.001
Anger	1085.13 ± 205.35	1024.34 ± 199.12	916.72 ± 150.04	0.009
Happiness	641.57 ± 165.74	683.29 ± 200.52	519.51 ± 90.14	0.002
Surprise	982.64 ± 204.99	905.99 ± 197.96	775.99 ± 165.86	0.001
Fear	1090.99 ± 186.53	1000.80 ± 245.67	889.76 ± 174.86	0.004
Disgust	990.93 ± 183.91	959.86 ± 213.00	834.35 ± 174.97	0.014

**FIGURE 4:**
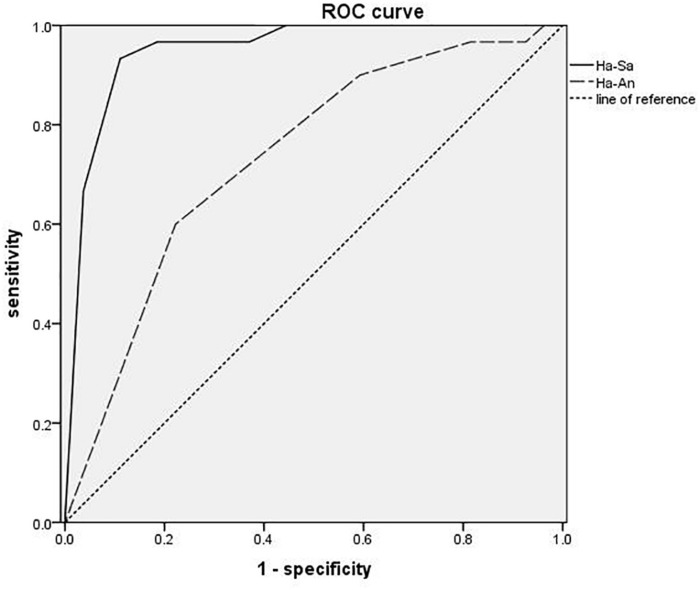
ROC curve of distinguish unipolar and bipolar depression.

The ROC analysis is shown in Figure 4, using happiness-sadness to distinguish unipolar and BDs, the area under the ROC curve (AUC) is 0.933, the maximum Youden index is 0.822, the specificity is 0.889, and the sensitivity is 0.667. Using happiness-anger to distinguish unipolar and BD, the AUC was 0.733, the maximum Youden index was 0.378, the specificity was 0.778, and the sensitivity was 0.600.

## Discussion

Bipolar disorder is common with depressive episodes, resulting in a very similar appearance to UD, but they differ greatly in etiology, course of disease, and course of treatment (Mustafa et al., 2019). Therefore, it is particularly important to find a way to identify the two diseases. This experiment found that in the UD group and BD group compared with the normal control group, the recognition ability of facial expression is generally reduced. This is consistent with the results of many previous studies. As the cognitive function of UD and BD patients declines, facial expression recognition ability declines (Kadlecova et al., 2013; Neves et al., 2015). Second, this study found that compared with the normal control group and UD group, patients with BD had a lower ability and longer recognition time to recognize happy expressions. This is similar to the findings of Lawlor-Savage et al. (2014), under time constraints, the ability of patients with bipolar disorder to recognize happiness expression decreases. However, unlike the results of Kjærstad et al. (2015), it is found that in social settings, patients with BD have a reduced ability to recognize negative expressions. This may be caused by the small number of participants.

In addition, this study found that compared with the two patient groups, the UD patient group had a longer time to recognize the negative expression, and the BD patient group had a longer time to recognize the happy expression. Although there is no statistical significance, it reflects from the side that UD patients may have a decreased ability to recognize negative expressions, and BD patients have a decreased ability to recognize positive expressions. Mendlewicz et al. (2005) have found that depressed people have a lower response to positive emotions and an impaired ability to experience them. Our experiments found that both groups of patients had impaired ability to respond quickly to recognize facial expressions, and BD patients had a lower react ability to experience positive emotions than UD patients. Vederman et al. (2012) found that patients with BD had a lower ability to recognize sad and fearful facial expressions than patients with UD, and inferred that this might be one of the characteristics of bipolar disorder. This experiment found that the ability of BD patients to recognize happy expressions decreased significantly, which may be caused by different cognitive paradigms, or it may be caused by less errors of enrolled subjects. In addition, this study also found that the difference of happy-sadness and happy-anger recognition in distinguishing UD and BD has high specificity, which also provides us with a new idea for further research.

During facial expression recognition, the ability to distinguish between two negative expressions or between negative and neutral expressions was decreased in both patients and controls. Some research suggests that fear and surprise should be considered part of the same emotional category at an adaptive level (Jack et al., 2014; Gordillo et al., 2017). Other studies also believe that surprise is a reaction to unexpected events and uncertain events. Accordingly, its effectiveness as a primary means of identifying fear is expected to diminish when it is associated with an emotional visual environment (Vrticka et al., 2014). This means that the distinction between expressions may be reflected in the basic brain activity patterns of facial expression recognition, or the brain regions that govern their expression come from the same region.

Psychotic patients have defects in recognizing facial emotions. However, the nature and extent of these changes are not fully understood. Therefore, it may also be useful to explore corresponding anatomical correlation studies to understand the evidence of existing neurological functions based on psychopathological features of neurodevelopmental roots. Morphological changes in brain regions play an important role in emotions and social cognition. Allegedly, the frontal and temporal lobes of the brain, insula, and amygdala are associated with neurodevelopment in psychotic patients (Leppänen and Nelson, 2006; Sethi et al., 2015; Pera-Guardiola et al., 2016). With the rapid development of neuroimaging technology, functional magnetic resonance imaging technology has been used as an important method for brain functional imaging research. This technique uses non-invasive imaging methods to combine high-resolution structural imaging technology with hemodynamics. The combination of neuroscience and brain activity can detect changes in the brain’s physiological structure (O’Connor et al., 2019). Early research believed that the amygdala played a very important role in the recognition of fear expressions. Later, it was found that the amygdala is involved not only in the recognition of fear but also in the recognition of other expressions such as happiness and sadness. Studies have also found that putamen can affect the recognition of feared faces, and work in conjunction with the amygdala to play an important role in the network model of emotion processing (Phan et al., 2002; Uono et al., 2017; Horne and Norbury, 2018). Previously, studies have found that insular damage is associated with a decline in the ability to recognize disgust, and it is believed that the insular-striatum system may be involved in all channels of disgust (Calder et al., 2000). Recent studies have also found that the insular plays an important role in the experience, expression, and recognition of disgust expressions, and the metabolic level of the insular is positively correlated with the feeling of disgust (Ory et al., 2017; Holtmann et al., 2020). Combined with this study, it is speculated that patients with UD and BD may have differences in the brain functional areas corresponding to positive and negative facial expressions, such as amygdala, putaminara, and insula.

Through comparing facial expression recognition of patients with UD and BD disorders and the control group, along with neuropsychology and neuroimaging, our experiment proposed a conjecture and hypothesis on the differences in the functional brain regions of patients with UD and BD. Unlike many paradigms, choosing from a limited set of two expressions greatly improves the accuracy of patient recognition. Each patient identified six facial expressions 300 times, which greatly increases the credibility of the experiment. However, this study is not without shortcomings. First, the sample size of the study is small; thus, it is necessary to further increase the sample size to increase the reliability of the results. The accuracy of facial expression recognition is influenced by many factors, such as the time the stimulus is presented and the intensity of the expression. These objective factors are still a research area that requires continual exploration. Second, although we require UD patients enrolled to have at least two depressive symptoms, we cannot rule out the possibility that patients with BD are in the depressive phase. In future experiments, we should follow up patients regularly to see if they become mania and try to minimize the test error. In the future, we hope to provide stronger evidence for the difference between UD and BD by combining behavioral and brain functional imaging. At the same time, we expect to find biomarkers to distinguish UD from BD as soon as possible, so as to provide effective criteria for clinical differentiation of UD and BD.

Our data were collected from July 2018 to August 2019, when DSM-5 diagnostic criteria were not yet widely available in China, so DSM-IV diagnostic criteria were selected. In the DSM-IV and DSM-5, most of the major depressive disorder (MDD) criteria are the same. There are three variations to the MDD standard. First, the statement that inconsistent emotional delusions or hallucinations should not count toward an episode of major depression (MDE)/MDD diagnosis was removed. Second, the word “hopeless” was added to the subjective description of depressed mood. A subject who reported feeling hopeless but not sad met the DSM-5 emotional criteria, but not the DSM-IV emotional criteria. Third, the DSM-5 removes the “exclusion of bereavement” in the diagnosis of MDE and replaces it with a statement requiring clinical judgment when diagnosing MDD in the context of significant loss (Uher et al., 2014). Although there are some changes in the DSM-5 diagnosis of depression, the effect on this study was not significant. In the future, patients will be admitted according to the latest diagnosis of depression.

## Conclusion

Patients with UD had lower performance in recognizing negative expressions and had longer recognition times. Those with BD had lower accuracy in recognizing positive expressions and longer recognition times. This study provides preliminary behavioral evidence for the differentiation of UD and BD, and suggests that rapid facial expression recognition may be a potential endophenotype.

## Data Availability Statement

The original contributions presented in the study are included in the article/supplementary material; further inquiries can be directed to the corresponding author/s.

## Ethics Statement

The studies involving human participants were reviewed and approved by the Research Ethics Committee at the Beijing Huilongguan Hospital. The patients/participants provided their written informed consent to participate in this study. Written informed consent was obtained from the individual(s) for the publication of any potentially identifiable images or data included in this article.

## Author Contributions

WZ and ZK developed the concept and design of this study. MR and ZM performed the experiments and analyzed the data. CN, GH, LP, LS, TS, TY, SJ, TL, and YF restructured, polished, and revised the manuscript. All authors contributed to the article and approved the submitted version.

## Conflict of Interest

The authors declare that the research was conducted in the absence of any commercial or financial relationships that could be construed as a potential conflict of interest.
